# RJunBase: a database of RNA splice junctions in human normal and cancerous tissues

**DOI:** 10.1093/nar/gkaa1056

**Published:** 2020-11-12

**Authors:** Qin Li, Hongyan Lai, Yuchen Li, Bing Chen, Siyuan Chen, Yan Li, Zhaohui Huang, Zhiqiang Meng, Peng Wang, Zhixiang Hu, Shenglin Huang

**Affiliations:** Department of Integrative Oncology, Fudan University Shanghai Cancer Center, and the Shanghai Key Laboratory of Medical Epigenetics, the International Co-laboratory of Medical Epigenetics and Metabolism, Ministry of Science and Technology, Institutes of Biomedical Sciences, Fudan University, Shanghai, China; Department of Oncology, Shanghai Medical College, Fudan University, Shanghai, China; Department of Integrative Oncology, Fudan University Shanghai Cancer Center, and the Shanghai Key Laboratory of Medical Epigenetics, the International Co-laboratory of Medical Epigenetics and Metabolism, Ministry of Science and Technology, Institutes of Biomedical Sciences, Fudan University, Shanghai, China; Department of Oncology, Shanghai Medical College, Fudan University, Shanghai, China; Department of Integrative Oncology, Fudan University Shanghai Cancer Center, and the Shanghai Key Laboratory of Medical Epigenetics, the International Co-laboratory of Medical Epigenetics and Metabolism, Ministry of Science and Technology, Institutes of Biomedical Sciences, Fudan University, Shanghai, China; Department of Oncology, Shanghai Medical College, Fudan University, Shanghai, China; Department of Integrative Oncology, Fudan University Shanghai Cancer Center, and the Shanghai Key Laboratory of Medical Epigenetics, the International Co-laboratory of Medical Epigenetics and Metabolism, Ministry of Science and Technology, Institutes of Biomedical Sciences, Fudan University, Shanghai, China; Department of Oncology, Shanghai Medical College, Fudan University, Shanghai, China; Department of Oncology, Shanghai Medical College, Fudan University, Shanghai, China; Department of Integrative Oncology, Fudan University Shanghai Cancer Center, and the Shanghai Key Laboratory of Medical Epigenetics, the International Co-laboratory of Medical Epigenetics and Metabolism, Ministry of Science and Technology, Institutes of Biomedical Sciences, Fudan University, Shanghai, China; Department of Oncology, Shanghai Medical College, Fudan University, Shanghai, China; Wuxi Cancer Institute, Affiliated Hospital of Jiangnan University, Wuxi, China; Department of Integrative Oncology, Fudan University Shanghai Cancer Center, and the Shanghai Key Laboratory of Medical Epigenetics, the International Co-laboratory of Medical Epigenetics and Metabolism, Ministry of Science and Technology, Institutes of Biomedical Sciences, Fudan University, Shanghai, China; Department of Oncology, Shanghai Medical College, Fudan University, Shanghai, China; Department of Integrative Oncology, Fudan University Shanghai Cancer Center, and the Shanghai Key Laboratory of Medical Epigenetics, the International Co-laboratory of Medical Epigenetics and Metabolism, Ministry of Science and Technology, Institutes of Biomedical Sciences, Fudan University, Shanghai, China; Department of Oncology, Shanghai Medical College, Fudan University, Shanghai, China; Department of Integrative Oncology, Fudan University Shanghai Cancer Center, and the Shanghai Key Laboratory of Medical Epigenetics, the International Co-laboratory of Medical Epigenetics and Metabolism, Ministry of Science and Technology, Institutes of Biomedical Sciences, Fudan University, Shanghai, China; Department of Oncology, Shanghai Medical College, Fudan University, Shanghai, China; Department of Integrative Oncology, Fudan University Shanghai Cancer Center, and the Shanghai Key Laboratory of Medical Epigenetics, the International Co-laboratory of Medical Epigenetics and Metabolism, Ministry of Science and Technology, Institutes of Biomedical Sciences, Fudan University, Shanghai, China; Department of Oncology, Shanghai Medical College, Fudan University, Shanghai, China

## Abstract

Splicing is an essential step of RNA processing for multi-exon genes, in which introns are removed from a precursor RNA, thereby producing mature RNAs containing splice junctions. Here, we develope the RJunBase (www.RJunBase.org), a web-accessible database of three types of RNA splice junctions (linear, back-splice, and fusion junctions) that are derived from RNA-seq data of non-cancerous and cancerous tissues. The RJunBase aims to integrate and characterize all RNA splice junctions of both healthy or pathological human cells and tissues. This new database facilitates the visualization of the gene-level splicing pattern and the junction-level expression profile, as well as the demonstration of unannotated and tumor-specific junctions. The first release of RJunBase contains 682 017 linear junctions, 225 949 back-splice junctions and 34 733 fusion junctions across 18 084 non-cancerous and 11 540 cancerous samples. RJunBase can aid researchers in discovering new splicing-associated targets and provide insights into the identification and assessment of potential neoepitopes for cancer treatment.

## INTRODUCTION

The majority of human pre-mRNAs contain more than one exon and the process of pre-mRNA splicing, which removes introns and joins exons, is crucial for the maturation of functional mRNAs (1,2). Alternative splicing (AS) is a key factor of enlarging the transcriptome and proteome, allowing mammalian cells to generate distinct protein isoforms from a single gene. The splicing process is accomplished by a megadalton complex of RNAs and proteins that comprises five small nuclear ribonucleoprotein particles (snRNPs; U1, U2, U4, U5 and U6) and >200 splicing regulators (2). High-throughput sequencing-based methods and their applications in the study of transcriptomes have revolutionized our understanding of RNA splicing in a large number of diseases, especially in human cancers (3). Recent studies on transcriptomic profiling indicated that dysregulated splicing events are widespread in human cancers (4,5), and tumor-specific AS events can contribute to the initiation and progression of cancer, representing potential targets for cancer treatment (6,7). For example, Li *et al.* demonstrated that a splicing switch from ketohexokinase-C to ketohexokinase-A could drive hepatocellular carcinoma formation, which is dependent on c-Myc and heterogeneous nuclear ribonucleoproteins (8). Furthermore, based on the analysis of RNA splice junctions of tumor cells and tissues, tumor-specific transcripts (TSTs) have been shown to be overexpressed in several malignant tumors (9,10). These newly identified TSTs, such as LIN28B-TST and TST1, play key roles in carcinogenesis, and their high expression is correlated with worse overall survival of cancer patients (10,11). Moreover, the tumor-specific neoepitopes derived from splice junctions can represent promising targets for immunotherapy (12). Therefore, knowledge advances in these research fields have emphasized the need for a coordinated, community-based effort to construct public data resources that can systematically annotate and integrate important information regarding splicing patterns and functions. A compendium of these splice junctions will aid researchers in deciphering tumor heterogeneity at the junction level and support the discovery of new splicing-related targets.

To date, several RNA splicing databases have been developed with different purposes. For example, an exon-exon junction database (JuncDB, http://juncdb.carmelab.huji.ac.il/) was established for the comparison of architectures between orthologous transcripts across 88 eukaryotic species (13). Nellore *et al.* aligned 21,504 Illumina-sequenced human RNA samples and found 56 861 unannotated novel junctions, which were made available on the intropolis database (http://intropolis.rail.bio) (14). In the scope of human cancers, Xia *et al.* created the CSCD database (http://gb.whu.edu.cn/CSCD) to provide a visual interface for exploring the function and regulation of cancer-specific circRNAs based on cancer cell lines (15). Additionally, the FusionGDB database (https://ccsm.uth.edu/FusionGDB) also offers an integrative resource of gene fusions, including cancer-associated transcript fusions across all cancers (16). Ryan *et al.* developed the TCGASpliceSeq database (http://bioinformatics.mdanderson.org/TCGASpliceSeq) for exploring the AS patterns of The Cancer Genome Atlas (TCGA) tumors (17). Although these databases offer insightful information on RNA splicing, there is still a lack of a convenient database for analyzing various forms of splice junctions (linear, back-splice, and fusion junctions), potentially tumor-specific junctions, and unannotated junctions in human tissues.

In this study, we developed a web-accessible database, named RJunBase, to provide information on three types of RNA splice junctions that are derived from RNA-seq data of various non-cancerous and cancerous samples. This tool aims to help users search for their desired targets and obtain information about their splice patterns and expression profile. The whole dataset was designed to be easily browsed, queried, and downloaded through its webpage. In addition, RJunBase is expected to allow researchers submit new splice junctions and their expression profiles of human samples.

## MATERIALS AND METHODS

### Identification of linear junctions

We collected RNA-seq data of 10 283 samples containing primary, metastatic, and normal tissues across 33 cancer types from TCGA and obtained linear junction data from datasets stored at the Genotype-Tissue Expression (GTEx) portal (18). Metastatic and primary samples were considered to be in the same class as cancer samples. TCGA BAM files were aligned to the GRCh38 reference genome using a two-pass method with STAR algorithm. StringTie (version 1.2.3) was used to assemble the BAM files for all samples (19). Linear junctions were identified, quantified, and normalized based on the StringTie output file using the Assembling Splice Junctions Analysis (ASJA) software package, which identifies and characterizes all splice junctions from high-throughput RNA-seq data (9). Raw count data obtained from GTEx were normalized according to: raw_counts/sum_counts × 10 000 000 (sum_counts represent the sum of the counts of all samples), with the scale factor being the same as that adopted by ASJA. We removed all junctions that were present in fewer than two samples of GTEx and TCGA. Gene annotations were downloaded in the GFF/GTF format from GENCODE database (release 29, https://www.gencodegenes.org). Linear junctions were matched onto gene coordinates using the genomic intersection functionality of BEDTools (https://bedtools.readthedocs.io/) and were further classified as intragenic or intergenic junctions. All junctions were annotated with gene information, and junctions in the intergenic region were annotated with information of the downstream gene. In addition, oncogene and tumor suppressor gene annotations were derived from ONGene (20) and TSGene (21), and were pooled together and named tumor-associated genes. Junctions from tumor-associated genes were labeled as ‘yes’ in ‘Tumor associated genes’ column in the RJunBase. Five types of alternative splice junctions (intron retention, exon skipping, mutually exclusive exons, alternative 3′, and alternative 5′ splice) were extracted from a paper published by André Kahles *et al.* (4). The genomic coordinates of alternative splice junctions were converted from hg37 to hg38 using the University of California Santa Cruz (UCSC) LiftOver tool.

The unannotated junctions were defined as the junctions that are not annotated in GENCODE. To obtain high-confidence unannotated junctions, we removed those unannotated junctions that were expressed in <10 samples. The majority of the unannotated junctions (89.45%) contained the canonical splice site (GT-AG).

The junctions were identified as tumor-specific junctions when the following criteria were met: NT_max = 0 & nTE_max = 0 & Freq_tumor > 5%; or Tumor_median > 10 * NT_max & Tumor_median > 10 * nTE_max & Freq_tumor > 5%. Tumor_median is the median expression value of tumor samples, NT_max is the maximal expression value of TCGA normal samples, nTE_max is the maximal expression value of GTEx normal tissues, and Freq_tumor is the frequency of junctions expressed in the cancer.

### Collection of back-splice and fusion junctions

The location and expression information of back-splice junctions from over 2000 samples across 27 cancer types were obtained from MiOncoCirc database (https://mioncocirc.github.io/) (22). The total number of back-splice reads for every sample was calculated. The majority of these samples contained 1200–730 000 back-splice reads. Only 37 samples contained <1000 back-splice reads, which were considered to be low-quality data. Therefore, they were filtered to prevent any impact on the subsequent analyses. MiOncoCirc included 25 normal samples, which were removed and not included in the RJunBase for the extreme imbalance between cancer and normal sample sets. Samples with ambiguous classification were also removed. The novel read-through circRNA junctions involving two different genes were not included in the RJunBase because of the lack of detailed expression data.

Fusion junctions were downloaded from a previous report published by Dehghannasiri *et al.* (23). They developed the DataEnriched Efficient PrEcise STatistical (DEEPEST) fusion detection algorithm (https://github.com/salzmanlab/DEEPEST-Fusion) to detect gene fusions emerging at annotated exon boundaries. This algorithm used RNA-seq fastq files from 9946 TCGA samples as input data and identified over 30 000 fusions. Gene information for the two types of junctions in the RJunBase was then annotated based on the GENCODE database (release 29). The expression of fusion junctions were standardized as linear junctions according to: raw_counts/sum_cov × 10 000 000 (sum_cov: sum coverage of samples). The expression frequency (and corresponding sample number) and median value in cancer cohorts were calculated for each back-splice and fusion junction.

### Visualization of the splicing pattern and expression profile

We used a custom python-based script to plot all relevant forward- and back-splice junctions for each gene. Hosting genes of fusion junctions were efficiently and flexibly visualized in a circular layout using the R software package ‘circlize’ (24), and the gene length was scaled to 100 bp to facilitate graphical representation. The expression profile was depicted using the ‘ggplot2’ R package. We stratified samples in the same cancer cohort into two groups (high and low expression groups) under the median (or mean) of junction expression values. The Kaplan–Meier survival curves were plotted using ‘ggplot2’.

### Names of the three junctions types

A unique name was provided for all junctions. Linear, fusion, and back-splice junction types were named as LS, FS, BS, respectively. Unannotated junctions were named as UN. UP means junction locate in the upstream region of neighbor genes. Annotated linear junctions in the intragenic region were named as Genename_LS+ID, where Genename is the hosting gene of the junction and ID is the rank number of the annotated junctions according to genomic location for that hosting gene. Unannotated linear junctions in intragenic regions were named as UN_Genename_LS+ID, where ID is the counterpart of the unannotated junctions for that hosting gene. Linear junctions in intergenic regions were named as UN_Genename_LS_UP+ID, where Genename is the downstream gene of the junction, and ID is the rank number of unannotated junctions according to genomic location. Intergenic junctions were annotated with information of downstream genes as a large part of these junctions was generated by alternative promoters, which are located in the upstream region of neighbor genes. A similar phenomenon has been reported by Demircioğlu *et al.* (5). Back-splice junctions were named as Genename_BS+ID. Fusion junctions were named as Genename1-Genename2_FS+ID, where Genename1 and Genename2 are the two hosting genes of the junction.

### Database implementation

The RJunBase was built using MySQL (database server) and Akka 2.6.3 (web server). All data were handled and organized into a MySQL Database Management System (version 5.7). The interface of the website was designed and achieved using the Twirl template engine. The query system for the database was based on Scala script. The website was tested in several distinct popular web browsers such as Google Chrome, Firefox, and Internet Explorer.

## RESULTS

### Data summary

**Figure 1.**
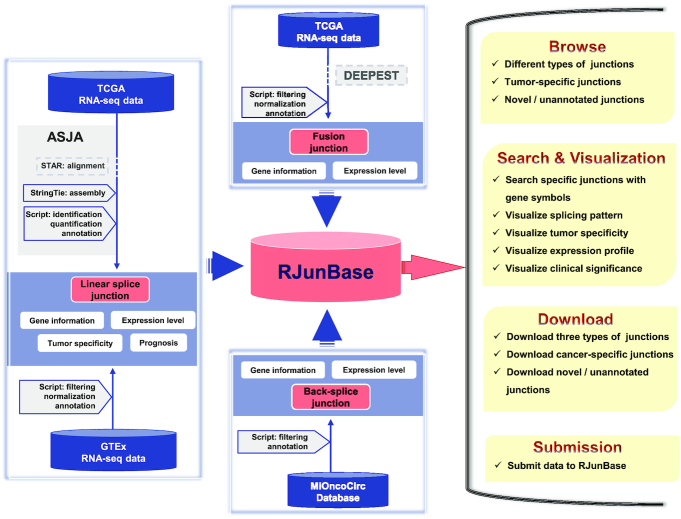
Data generation flow of RJunBase. RJunBase collects and extracts linear, back-splice, and fusion junctions from available data resources. The linear junctions in cancer tissues were extracted from the Cancer Genome Atlas (TCGA) BAM files using the Assembling Splice Junctions Analysis (ASJA) software package. The linear junctions in normal tissues were directly collected from the Genotype-Tissue Expression (GTEx) database. Fusion junctions were derived from Dehghannasiri *et al.* study, which was based on TCGA RNA-seq data and the DataEnriched Efficient PrEcise STatistical fusion detection (DEEPEST) algorithm. Back-spliced junctions were obtained from the MiOncoCirc database. All splice junctions in RJunBase were quantified and annotated. Contents of the RJunBase database include Browse, Search and Visualization, Downloads, and Submission.

RJunBase collects and integrates three types of splice junctions from different non-cancerous and cancerous human tissues (Figure 1). The current version of RJunBase comprises 682 017 linear junctions, 225 949 back-splice junctions and 34 733 fusion junctions, covering a total of 29 624 samples from the TCGA (10 283 samples), GTEx (17 382 samples) and MiOncoCirc (1959 samples) databases. RJunBase also includes 324 651 unannotated junctions and 32 837 tumor-specific junctions. The results of the detected linear and fusion junctions from TCGA samples and the linear junctions from GTEx are summarized in Tables 1 and 2, respectively. The numbers of back-splice junctions are summarized in Table 3. The Supplementary Table S1 provides the abbreviations and full names for all 47 cancer types included in the RJunBase database.

**Table 1.** Numbers of linear junctions and fusion junctions from the Cancer Genome Atlas

Cancer type	Sample number^a^	Cancer number^b^	NT number^c^	LJ number^d^	FJ number^e^	AJ number^f^	UN number^g^
ACC	79	79	0	167 995	334	133 087	34 907
BLCA	433	414	19	414 850	1 588	215 258	199 591
BRCA	552	458	94	465 831	6 935	222 871	242 959
CESC	306	306	0	346 688	573	197 634	149 053
CHOL	45	36	9	156 137	80	129 851	26 285
COAD	518	480	38	381 256	355	189 084	192 171
DLBC	48	48	0	147 193	55	117 191	30 001
ESCA	172	162	10	444 738	1 609	156 997	287 740
GBM	172	167	5	299 625	651	180 052	119 572
HNSC	546	502	44	412 916	986	210 633	202 282
KICH	89	65	24	203 201	42	151 461	51 739
KIRC	607	535	72	442 970	421	216 856	226 113
KIRP	321	289	32	317 241	270	190 222	127 018
LAML	151	151	0	266 377	166	140 441	125 935
LGG	479	479	0	352 478	997	193 561	158 916
LIHC	423	373	50	360 938	944	187 007	173 930
LUAD	585	526	59	463 878	1 962	223 334	240 543
LUSC	527	478	49	473 335	1 895	225 184	248 150
MESO	86	86	0	192 064	170	146 617	45 446
OV	346	346	0	517 418	3 368	116 259	401 158
PAAD	182	178	4	289 682	223	179 089	110 592
PCPG	186	183	3	227 760	167	157 069	70 690
PRAD	552	500	52	368 670	2 045	197 897	170 772
READ	177	167	10	214 140	175	151 440	62 699
SARC	265	263	2	335 523	2 885	198 122	137 400
SKCM	472	471	1	395 984	1 724	208 472	187 511
STAD	405	373	32	544 749	2 846	117 382	427 366
TGCT	156	156	0	278 687	106	183 344	95 342
THCA	561	503	58	365 678	275	194 155	171 522
THYM	119	119	0	235 115	64	161 858	73 256
UCEC	587	552	35	421 263	1 002	209 023	212 239
UCS	56	56	0	193 121	443	149 924	43 196
UVM	80	80	0	148 517	37	121 894	26 622

ACC, adrenocortical carcinoma; BLCA, bladder urothelial carcinoma; BRCA, breast invasive carcinoma; CESC, cervical squamous cell carcinoma and endocervical adenocarcinoma; CHOL, cholangiocarcinoma; COAD, colon adenocarcinoma; DLBC, lymphoid neoplasm diffuse large B-cell Lymphoma; ESCA, esophageal carcinoma; GBM, glioblastoma multiforme; HNSC, head and neck squamous cell carcinoma; KICH, kidney chromophobe; KIRC, kidney renal clear cell carcinoma; KIRP, kidney renal papillary cell carcinoma; LAML, acute myeloid leukemia; LGG, brain lower grade glioma; LIHC, liver hepatocellular carcinoma; LUAD, Lung adenocarcinoma; LUSC, lung squamous cell carcinoma; MESO, mesothelioma; OV, ovarian serous cystadenocarcinoma; PAAD, pancreatic adenocarcinoma; PCPG, pheochromocytoma and paraganglioma; PRAD, prostate adenocarcinoma; READ, rectum adenocarcinoma; SARC, sarcoma; SKCM, skin cutaneous melanoma; STAD, stomach adenocarcinoma; TGCT, testicular germ cell tumors; THCA, thyroid carcinoma; THYM, thymoma; UCEC, uterine corpus endometrial carcinoma; UCS, uterine carcinosarcoma; UVM, uveal melanoma.

^a^Sample number: total number of samples studied for each cancer type.

^b^Cancer number: number of cancer samples.

^c^NT number: number of paracancer samples.

^d^LJ number: number of linear junctions extracted from each cancer type.

^e^FJ number: number of fusion junctions extracted from each cancer type.

^f^AJ number: number of annotated junctions in linear junctions.

^g^UN number^:^ number of unannotated junctions in linear junctions.

**Table 2.** Numbers of linear junctions from the Genotype-Tissue Expression database

Tissue	Sample number	Linear junction number
Blood	929	314 347
Stomach	359	313 525
Colon	779	321 214
Pancreas	328	307 163
Skin	1809	326 671
Vagina	156	305 627
Uterus	142	301 319
Adipose tissue	1204	323 487
Fallopian Tube	9	256 858
Cervix uteri	19	270 935
Adrenal gland	258	308 438
Esophagus	1445	324 617
Thyroid	653	320 631
Prostate	245	315 279
Brain	2 642	328 652
Salivary gland	162	307 159
Pituitary	283	316 455
Muscle	803	315 604
Spleen	241	305 618
Breast	459	319 900
Kidney	89	298 646
Blood vessel	1335	322 300
Nerve	619	318 429
Liver	226	299 223
Bladder	21	271 004
Heart	861	317 675
Lung	578	319 823
Testis	361	329 431
Ovary	180	304 751
Small intestine	187	310 582

**Table 3.** Numbers of back-splice junctions from the MiOncoCirc database

Cancer type	Sample number	BS^a^ junction number
ACC	24	29 254
ALL	21	50 900
AML	10	31 068
BLCA	49	44 714
BRCA	236	98 906
CHOL	62	50 133
COLO	29	27 875
ESCA	19	34 621
GBM	26	34 245
HCC	17	37 275
HNSC	49	40 095
KDNY	23	31 254
LEUK	33	44 005
LUNG	40	35 738
MISC	124	74 556
MM	212	82 083
MPN	151	70 543
NHL	124	84 016
NRBL	13	27 659
OV	20	24 319
PAAD	58	44 155
PRAD	340	97 559
RHABDO	10	20 901
SARC	167	78 733
SECR	44	48 545
SKCM	31	28 810
STAD	27	30 775

ACC, adrenocortical carcinoma; ALL, acute lymphoblastic leukemia; AML, acute myeloid leukemia; BLCA, bladder urothelial carcinoma; BRCA, breast invasive carcinoma; CHOL, cholangiocarcinoma; COLO, colon cancer; ESCA, esophageal carcinoma; GBM, glioblastoma multiforme; HNSC, head and neck squamous cell carcinoma; KDNY, renal cell carcinoma; LEUK, leukemia; LUNG, lung adenocarcinoma; MISC, rare cancer; MM, multiple myeloma; MPN, myeloproliferative neoplasm; NHL, non-Hodgkin's lymphoma; NRBL, neuroblastoma; OV, ovarian serous cystadenocarcinoma; PAAD, pancreatic adenocarcinoma; PRAD, prostate adenocarcinoma; RHABDO, rhabdomyosarcoma; SARC, sarcoma; SECR, gandular cancer; SKCM, skin cutaneous melanoma; STAD, stomach adenocarcinoma.

^a^BS: back-splicing.

### Browse the database

**Figure 2.**
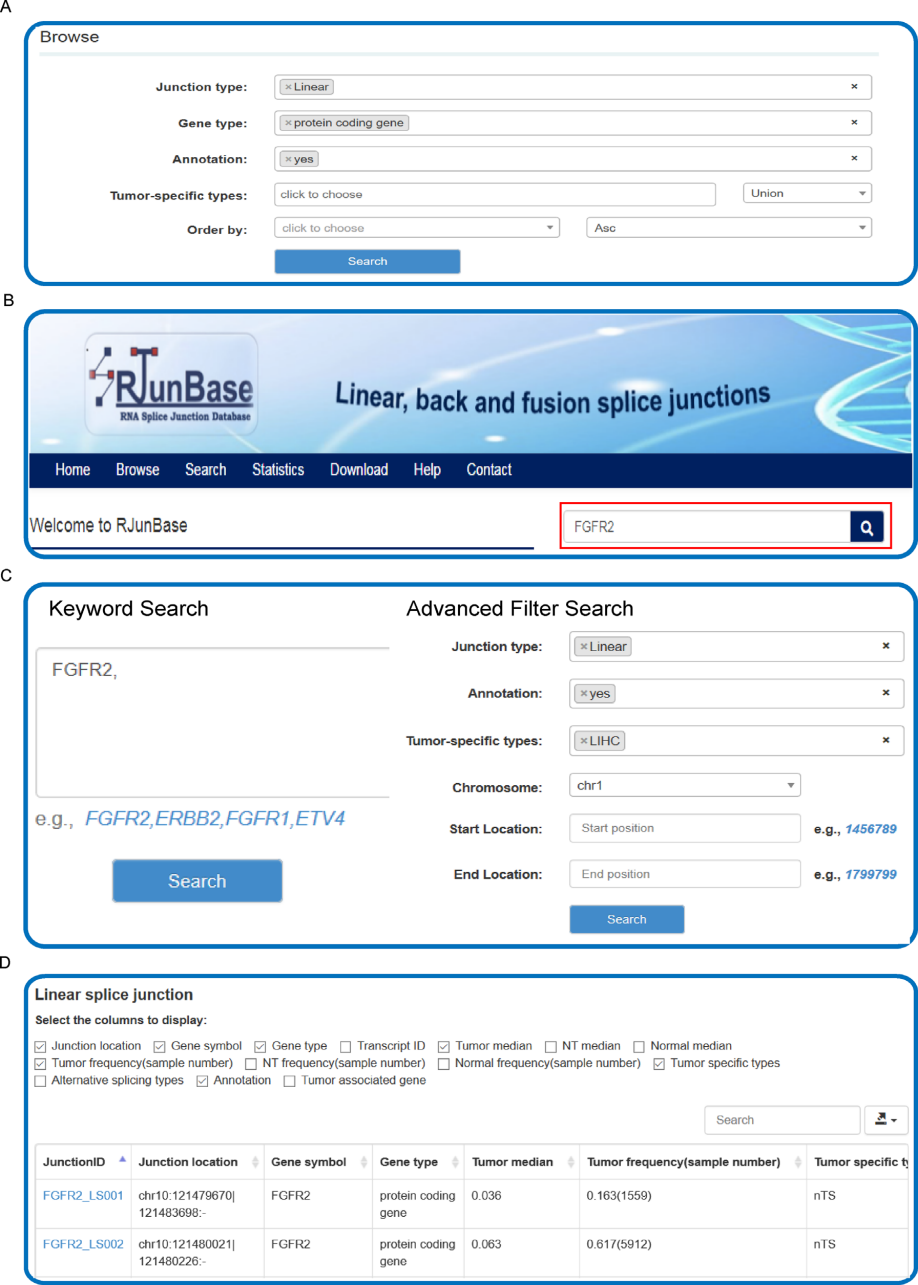
Interactive searching and browsing activity in RJunBase. (**A**) Browse database for splice junctions. (**B**) Quick search feature on the homepage. (**C**) The ‘Search’ page of RJunBase allows to search the database by the gene name or other options. (**D**) Results table for Browse or Search. ‘Tumor median’ and ‘NT median’ indicate the median expression value of cancer and adjacent normal tissues from the Cancer Genome Atlas (TCGA), respectively. ‘Normal median’ indicates median expression value of normal tissues from the Genotype-Tissue Expression (GTEx) database. ‘Tumor frequency(sample number)’ and ‘NT frequency(sample number)’ display the expression frequency of tumors and adjacent normal tissues from TCGA, respectively. ‘Normal frequency(sample number)’ displays the expression frequency of normal tissues from GTEx. ‘Tumor specific types’ indicates cancer types in which the junction are identified as tumor specific. ‘Alternative splicing types’ indicates types of alternative splicing of splice junctions.

Users can browse the information on junctions by clicking the ‘Browse’ tab on the left-side navigation menu, and the browse page will present the entry point to tables containing all the information on the three types of junctions (Figure 2A). Users can browse the RJunBase database with various options. For example, the ‘Junction type’ allows users to browse junctions of interest, such as fusion junctions. The ‘Gene type’ could be specified to select a particular type of hosting gene for each junction, such as ‘protein coding’. If not selected, junctions of all types will be displayed. For fusion junctions, the hosting genes, such as protein coding genes, are connected by ‘-’. Moreover, RJunBase provides two additional options to screen potentially functional junctions: (i) ‘Annotation’ indicates whether selected junctions can be extracted from GENCODE database; (ii) ‘Tumor-specific types’ denotes whether the junctions currently being browsed are tumor-specific. The RJunBase includes tumor-specific junctions that are related to 33 TCGA cancer types.

To obtain the analysis results only a click on the ‘Run’ button is necessary and a dynamic tabular with optional boxes will be displayed, in which each row and column show a junction and region-specific information respectively, including Junction ID, Junction location, Junction type, Gene symbol, Gene type, Tumor median, Tumor frequency (sample number), NT frequency (sample number), Tumor specific types, Annotation. Users can also obtain Transcript ID, NT median, Normal median, Normal frequency (sample number), and Alternative splicing types by clicking the corresponding menu options (Figure 2D). By default, each page displays 10 records and the user can view the remaining records through using the pagination features on the bottom right of the table. The number of records displayed in each page can be switched between 10, 25, 50 and 100 using the ‘records per page’ dropdown menu. Details about each junction can be viewed by clicking the junction ID, and clicking the Gene_symbol can direct users to the search results.

### Search the database

RJunBase provides a simple search interface and genes of interest can be easily queried (Figure 2B). Users can enter one (e.g., *ERBB2*) or more gene symbols that are separated by commas (e.g. *ETV4*, *FGFR2*) in the ‘Keyword Search’ field. Advanced filters, including Junction type, Annotation, Tumor-specific types, and Genomic location, allow users to further filter the results (Figure 2C). To obtain the basic information on all junctions related to the input gene, only a click on the ‘Search’ button is required. The ‘Search Results’ include a ‘Splicing pattern chart’ and responsive tables. The ‘Splicing pattern chart’ displays visualizations of the junctions that are contained in the input gene. By clicking in the ‘Linear&Back’ button, users will obtain a comprehensive plot in a publication-quality format of all linear and back-splice junctions, and the corresponding exon information from the reference gtf file will also be attached (25). A ‘Fusion’ button will be displayed if fusion junctions exist in the host gene, which will provide a Circos plot of fusion junctions between the two hosting genes. As for the responsive tables, which provide the annotation and expression information on the three types of junctions, clicking on the JunctionID hyperlinks will lead users to a page containing detailed information about the corresponding junctions.

### Detail page and result

Users can obtain basic junction annotations in the summary page. By clicking the ‘Expression profile’ button, the expression profile of the input junction across all cancer and normal tissues will be shown in boxplots. ‘Boxplot’ dynamically plots expression profiles of a given junction according to user-defined cancer selections and methods. The RJunBase also performs survival analysis based on junction expression profiles, allowing users to select their custom cancer types for overall or disease-free survival analysis. For example, to examine the survival curves of liver cancer patients based on the expression of the input junction in their cancer samples, users can select liver hepatocellular carcinoma (LIHC). RJunBase uses the Kaplan–Meier method for hypothesis evaluation, and the thresholds for high/low expression cohorts are adjustable.

### Applications of RJunBase: case 1

**Figure 3.**
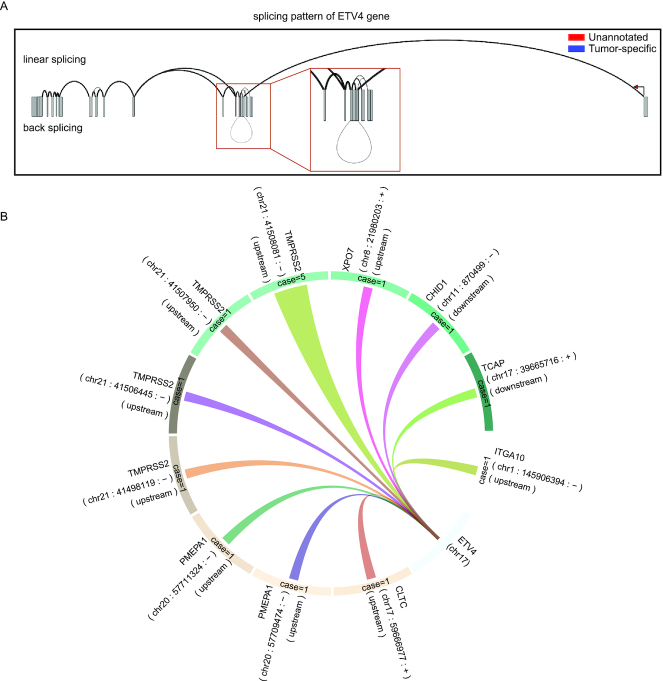
Examples of RJunBase outputs. (**A**) Users can check linear and back-splice junctions for *ETV4* in the ‘Linear&Back’ tab. (**B**) Circular layout of fusion junctions for *ETV4* in the ‘Fusion’ tab. A random color was assigned to links to distinguish between fusions. Case number of fusions was indicated in the circular ring, and the width was set to change with case number. Spatial information was displayed outside of the circular ring in reverse-clockwise manner.

The user can investigate splice junctions for desired genes in pan-cancer and normal tissues. For example, ETS variant transcription factor 4 (*ETV4*) holds a potential role in endometrial tumor-specific estrogen receptor binding (26). As a demonstration, we entered ‘ETV4’ in the search box on the home page or ‘Search’ page. The search results were classified into three parts: the splicing pattern chart, the table of three junctions, and the junction detail. The splicing pattern chart showed that *ETV4* contained linear junctions and back-splice junctions (Figure 3A). Furthermore, the circular layout showed genes fused to *ETV4* (Figure 3B). The search results also listed a table that contains linear, back-splice, and fusion junctions.

**Figure 4.**
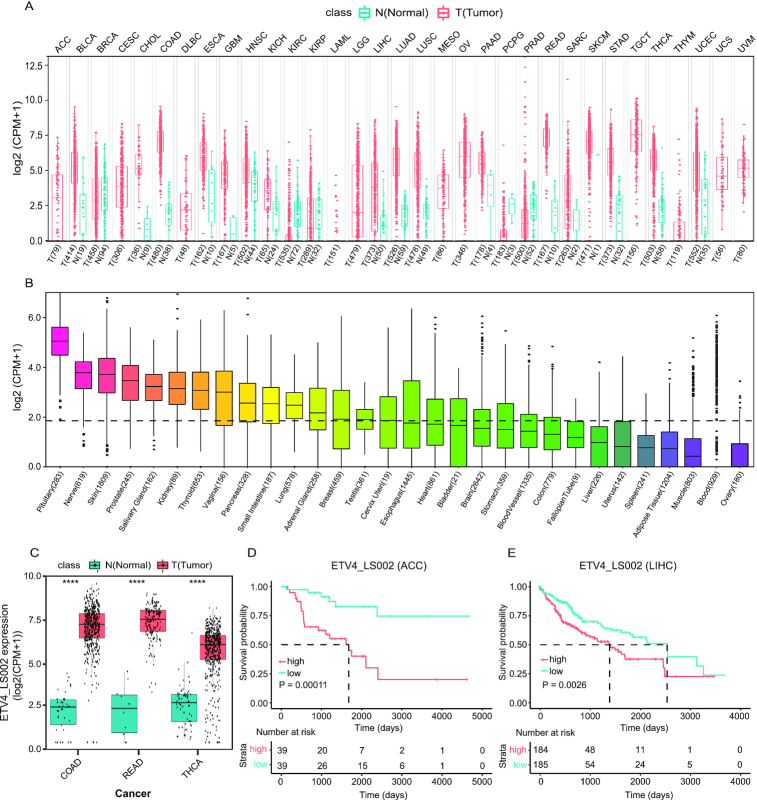
Expression profile and prognostic significance of ETV4_LS002 junction. (**A, B**) Expression of ETV4_LS002 in the Cancer Genome Atlas (TCGA) (**A**) and the Genotype-Tissue Expression (GTEx) database (**B**) samples in the ‘Summary’ tab on the ‘Linear Splice Junction Detail’ page. (**C**) Expression of ETV4_LS002 in COAD, READ, and THCA samples in the ‘Expression DIY’ tab. (**D, E**) Overall survival analysis of ETV4_LS002 junction in ACC (**D**) and LIHC (**E**) in the ‘Survival’ tab. Abbreviations: ACC, adrenocortical carcinoma; COAD, colon adenocarcinoma; LIHC, liver hepatocellular carcinoma; N, normal; READ, rectum adenocarcinoma; T, tumor; THCA, thyroid carcinoma.

Considering the median expression value and frequency of tumor, ETV4_LS002 was used as a test research subject by clicking ‘ETV4_LS002’ and switching to the junction detail page. The summary page showed that this specific junction existed in multiple transcripts, and its expression in cancer was much higher than in pan-cancer and normal tissues. Figure 4A and B shows the expression of this junction in pan-cancer and the GTEx database. Expression of this junction in colon adenocarcinoma, rectum adenocarcinoma, and thyroid carcinoma were presented individually in the ‘Expression DIY’ tab (Figure 4C). Survival curves were plotted for several cancers. High ‘ETV4_LS002’ expression was found to be correlated with decreased overall survival for adrenocortical carcinoma (Figure 4D) and liver hepatocellular carcinoma (LIHC) (Figure 4E).

### Applications of RJunBase: case 2

**Figure 5.**
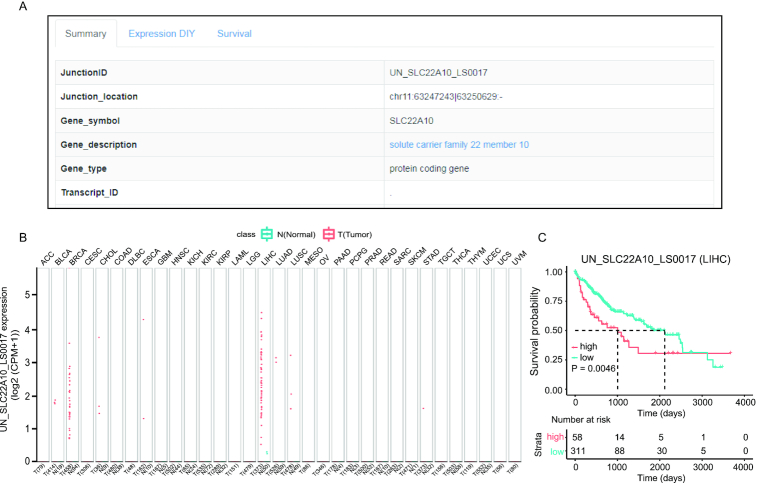
Example of tumor-specific junctions. (**A**) Summary of UN_SLC22A10_LS0017 junction. (**B**) Boxplot of normalized UN_SLC22A10_LS0017 expression profiles in pan-cancer. (**C**) Kaplan–Meier plot for overall survival of UN_SLC22A10_LS0017 junction in liver hepatocellular carcinoma (LIHC).

The potential functional implication of the RJunBase is to extract tumor-specific junctions. In this second situation, UN_SLC22A10_LS0017 was used as an example (Figure 5A), which is a tumor-specific junction in *SLC22A10*. As shown in Figure 5B, UN_SLC22A10_LS0017 was found to be highly expressed in LIHC and breast cancer. Survival analysis revealed that high expression of UN_SLC22A10_LS0017 was significantly associated with poorer prognosis in LIHC (Figure 5C). Therefore, UN_SLC22A10_LS0017 may function as an independent prognostic biomarker in patients with LIHC.

## DISCUSSION AND FUTURE DIRECTIONS

To the best of our knowledge, RJunBase is the first database of RNA splice junctions, which include linear, fusion and back-splice junctions from 11 540 cancerous and 18 084 non-cancerous human tissues. Compared with the TCGASpliceSeq database (17), RJunBase offers information on RNA splice junctions of cancer transcriptome that is not limited to AS patterns of protein-coding genes, and enables users to explore and visualize the gene-level splice pattern and the junction-level expression profile. This new database also supports users to assess desired splice junctions with custom query options, such as unannotated junctions and tumor-specific junctions. Additionally, researchers can download full datasets for integrative analysis, which will be beneficial in the search for new splicing-associated targets for cancer treatment.

In accordance with the existence of TSTs across human cancers, our database highlights the abundance of tumor-specific junctions. Tumor-specific junctions may be generated from multiple factors, such as epigenetic changes (chromatin remodeling or the activation of long terminal repeats) and mutations in cis-regulatory sequences or trans-acting splicing regulatory factors (27,28). For example, we previously depicted a TST of lin-28 homolog B, named LIN28B-TST (10). This TST was transcribed from an upstream long terminal repeat that possesses strong promoter activity with high-level H3K27Ac and H3K4me3 histone modifications, and an abundance of RNA polymerase II. The LIN28B-TST-specific junction could be browsed in the RJunBase database (LIN28B_LS001). Importantly, neojunctions possess the ability to form a class of potential neoantigens (4). These tumor-specific junctions may act as diagnostic markers and potentially as therapeutic targets for cancer patients.

We also discovered plenty of unannotated novel junctions in pan-cancer tissues and non-cancerous tissues, which could be divided into intergenic and intragenic junctions. The intergenic junctions may be generated by epigenetic alterations such as transcriptional activation, whereas the intragenic junctions may be produced by aberrant splicing in a single gene. It should be noted that some unannotated junctions may be by-products of the bioinformatics RNA splicing analysis. Recent advances in long-read sequencing identified full-length transcripts in different species (29–31). By using information on full-length transcripts and junctions from the RJunBase, users can further understand the splicing characteristics of specific targets to overcome identification of false-positive splice junctions.

The RJunBase will be regularly updated to continuously include more splice junctions of healthy and pathological human tissues, especially cancer tissues, including rare cancers. We believe that RJunBase will serve as an important resource and a powerful tool for studying splice junctions in human cancer transcriptomes and further understanding specific transcript production. Furthermore, the use of this database could potentially lead to the discovery of diagnostic and therapeutic targets for cancer.

## Supplementary Material

gkaa1056_Supplemental_File
