# Supplementary material for: RJunBase: a database of RNA splice junctions in human normal and cancerous tissues
Source: Nucleic Acids Res. 2020 Nov 12;49(D1):D201–11. doi: 10.1093/nar/gkaa1056 (PMC7779070; doi:10.1093/nar/gkaa1056)
Supplement: gkaa1056_Supplemental_File [file gkaa1056_supplemental_file.pdf]

Supporting material

RJunBase: a database of RNA splice junctions in human normal and cancerous tissues

Qin Li[1,3,4], Hongyan Lai[1,3,4], Yuchen Li[1,3], Bing Chen[1,3], Siyuan Chen[3], Yan Li[1,3], Zhaohui Huang[2], Zhiqiang Meng[1,3], Peng Wang[1,3], Zhixiang Hu[1,3,*], Shenglin Huang[1,3,*]

[1]Department of Integrative Oncology, Fudan University Shanghai Cancer Center, and the Shanghai Key Laboratory of Medical Epigenetics, the International Co-laboratory of Medical Epigenetics and Metabolism, Ministry of Science and Technology, Institutes of Biomedical Sciences, Fudan University, Shanghai, China

[2]Wuxi Cancer Institute, Affiliated Hospital of Jiangnan University, Wuxi, China

[3]Department of Oncology, Shanghai Medical College, Fudan University, Shanghai, China

[4]These authors contributed equally to this work

*** Correspondence:** Shenglin Huang, Ph.D., slhuang@fudan.edu.cn; or Zhixiang Hu, Ph.D., zhixiang211@126.com. Fudan University Shanghai Cancer Center and Institutes of Biomedical Sciences, Fudan University, 270 Dong An Rd., Shanghai 200032, China. Tel.: 86-21-34777580; Fax: 86-21-64172585.

Supplementary Table S1. The abbreviations and full names for all cancer types in RJunBase.

Cancer type (abbr.)	Cancer type (full name)
ACC	Adrenocortical Carcinoma
ALL	Acute Lymphoblastic Leukemia
AML	Acute Myeloid Leukemia
BLCA	Bladder Urothelial Carcinoma
BRCA	Breast Invasive Carcinoma
CESC	Cervical Squamous Cell Carcinoma and Endocervical Adenocarcinoma
CHOL	Cholangiocarcinoma
COAD	Colon Adenocarcinoma
COLO	Colon Cancer
DLBC	Lymphoid Neoplasm Diffuse Large B-cell Lymphoma
ESCA	Esophageal Carcinoma
GBM	Glioblastoma Multiforme
HCC	Liver Cancer
HNSC	Head and Neck Squamous Cell Carcinoma
KDNY	Renal Cell Carcinoma
KICH	Kidney Chromophobe
KIRC	Pan-kidney Cohort
KIRP	Kidney Renal Clear Cell Carcinoma
LAML	Acute Myeloid Leukemia
LEUK	Leukemia
LGG	Brain Lower Grade Glioma
LIHC	Liver Hepatocellular Carcinoma
LUAD	Lung Adenocarcinoma
LUNG	Lung Adenocarcinoma
LUSC	Lung Squamous Cell Carcinoma
MESO	Mesothelioma
MISC	Rare Cancer
MM	Multiple_Myeloma
MPN	Myeloproliferative Neoplasm
NHL	Non-Hodgkin Lymphoma
NRBL	Neuroblastoma
OV	Ovarian Serous Cystadenocarcinoma
PAAD	Pancreatic Adenocarcinoma
PCPG	Pheochromocytoma and Paraganglioma
PRAD	Prostate Adenocarcinoma
READ	Rectum Adenocarcinoma
RHABDO	Rhabdomyosarcoma
SARC	Sarcoma
SECR	Glandular Cancer
SKCM	Skin Cutaneous Melanoma

STAD	Stomach Adenocarcinoma
TGCT	Testicular Germ Cell Tumors
THCA	Thyroid Carcinoma
THYM	Thymoma
UCEC	Uterine Corpus Endometrial Carcinoma
UCS	Uterine Carcinosarcoma
UVM	Uveal Melanoma
